# Depression and suicidal ideation among undergraduates in state tertiary institutions in Lagos Nigeria

**DOI:** 10.1371/journal.pone.0284955

**Published:** 2023-04-26

**Authors:** Temitope ’Wunmi Ladi-Akinyemi, Adaeze Precious Okpue, Oluseyi Adetola Onigbinde, Ifeoma Peace Okafor, Babatunde Akodu, Kofoworola Odeyemi

**Affiliations:** 1 Department of Community Health and Primary Care, College of medicine, University of Lagos, Idi-Araba, Lagos State, Nigeria; 2 Department of Family Medicine, Lagos University Teaching Hospital, Idi-Araba, Lagos State, Nigeria; 3 Department of Community Health and Primary Care, Lagos University Teaching Hospital, Idi-Araba, Lagos State, Nigeria; Chinese Academy of Sciences, CHINA

## Abstract

**Background:**

Depression is a common mental illness affecting majority of the world’s population. However, evidence has shown that undergraduates are at an even higher risk compared to the general population, of developing depression due to the various challenges they face during that period. Suicide has been discovered to be the second leading cause of death among young people. Suicide ideation has been proven to be a predictor for not only suicide attempts but also completed suicides. Therefore, the aim of this study was to assess depression and suicidal ideation among undergraduates in the state tertiary institutions in Lagos, Nigeria.

**Method:**

This study was a descriptive, cross-sectional study carried out among undergraduates in two state tertiary institutions in Lagos, Nigeria using self-administered questionnaire. A total of 750 respondents were recruited using the multistage sampling technique. Data was analysed using SPSS version 27 and the level of significance was set at p -value < 0.05

**Results:**

The survey was conducted among undergraduates in the two state tertiary institutions in Lagos State: Lagos State University (48.3%) and Lagos State Polytechnic (51.7%). The mean age of the respondents was 21.5 (2.7) years. Majority of the respondents were females (54%), single (98.1%), Christians (70.3%), and the source of income of majority of the students was parents (72.8%). From the case vignette used in the questionnaire, 47.6% of the respondents were able to correctly identify depression. The prevalence of depression and suicidal ideation in this study was 22.5% and 21.6% respectively. Depression was statistically significantly associated with suicidal ideation (p < .001). Risk factors that were statistically significantly associated with depression and suicidal ideation were low self-esteem (p < .001), intake of recreational drugs (p < .001), alcohol dependence (p < .001), and positive history of bullying (p < .001).

**Conclusion and recommendations:**

The proportion of respondents with good knowledge of depression was not satisfactory. A strong relationship was found between depression and suicidal ideation indicating that people with depression are at a high risk of having suicidal ideation. Risk factors that were associated with depression and suicidal ideation were bullying, low self-esteem, intake of recreational drugs, alcohol dependence, poor academic performance, sexual assault and being hit by a partner. More works need to be done by the government and non-governmental organisations, school administration and parents to increase the awareness on the symptoms and manifestations of depression and reduce the burden created by some of the risk factors identified in this study to combat depression and suicidal ideation.

## Introduction

Depression is a common mental disorder estimated to affect 300 million people worldwide [1]. It is characterised by persistent feeling of sadness and inability to enjoy activities one normally enjoys, accompanied by inability to carry out daily activities for at least two weeks. Globally, 4.4% of the world’s population was estimated to be suffering from depression [1]. It can either be recurrent or long-standing and significantly impairs an individual’s ability to function and cope with daily life [1]. It is not the same as simple grief, bereavement or mourning mood, as these are appropriate emotional responses towards unwanted circumstances [2]. Depression is the leading cause of disability and is a major contributor to the total global burden of disease [3]. Although depression can be found across all age groups, it has been found that there are different and diverse manifestations of this mental disorder [4].

Evidence shows that university students are at a higher risk of having depression compared to the normal population [5]. This could be because they are a particularly vulnerable group due to the critical transition they undergo from adolescence to adulthood, in addition to facing one of the most stressful moments in a person’s life [6]. Factors such as female gender [7, 8], hopelessness about the future [7], academic and relationship difficulties [9], financial burden [10], low social support [11], post-traumatic stress disorder and sleeping problems [12], family problems [13], death of a family member [9], domestic violence [9], addiction to alcohol disorder and cigarettes [14], and substance abuse have been found to increase the risk of depression in students [15]. Depression, if ignored and not treated, can progress, worsen and lead to self-harm with the most severe end-result being suicide [1].

Suicidal ideation can be defined as having thoughts of committing suicide [16]. Suicidal ideation is an important predictor of suicide attempts and completed suicides, and is a marker of other mental health problems among the youth [17]. The World Health Organisation (WHO) reported that suicide is the second leading cause of death among 15–29 year olds worldwide, constituting a major public health problem [3]. Depression has been found in many studies to be associated with suicidal ideation [18–20]. A study done in Malaysia among adolescents, revealed that the prevalence of suicidal ideation was significantly much higher among the respondents with depression (55.8%) compared to those who were not depressed (14.4%) [19].

According to the WHO, 800,000 persons approximately die from suicide globally each year [21], and for each adult who died by suicide there may have been 20 other attempts at suicide [22]. Suicide accounted for 1.4% of all deaths worldwide, making it the 18^th^ leading cause of death in 2016 [22]. The WHO has shown that 79% of suicides occurred in low- and middle-income countries in 2016 [22]. The WHO reports that the age-standardized suicide rates in Nigeria for both sexes in 2016 was 17.3 per 100,000 with the rate being 17.5 per 100,000 in males and 17.1 per 100,000 in females [23].

Evidence suggests that college students have a higher risk of having suicidal thoughts and behaviours and this can be due to numerous developmental and psychosocial changes they experience [24]. These challenges include developing their identity, for example, choosing their careers, leaving their primary support system, transitioning from semi-dependence on parents to full-dependence and the financial burden of increasingly high college tuition [24]. While there may be other factors such as hopelessness that predispose to suicidal ideation, it has been discovered that among college students, depression is a very significant precondition for suicidal ideation [24].

In 2015, the prevalence of depression in Nigeria was estimated to be 3.9% of the country’s population [1]. Other studies on depression from Nigeria reveal varying results [4, 12, 14]. Most of the data on suicide are from developed countries with little data from developing countries like Nigeria. The social stigma associated with suicide and the fact that it is considered a “taboo” in most areas is one of the reasons that there is under-reporting of suicidal attempts and cases [25]. Nevertheless, studies have been done to determine the prevalence of suicidal ideation and attempts in Nigeria. One of such studies was done among youths in Southwest Nigeria and this revealed that suicidal ideation was present in 20% of the respondents with approximately 12% suicidal attempts in the previous year [26].

Depression has been found to be a predictor for suicidal ideation [17]. Undergraduates have been found to be a high-risk population for not only depression, but also suicidal ideation [5]. Studies have shown that knowledge of depression among students, both adolescents and undergraduates is inadequate [9, 27] and not a lot of research has been done on the link between depression and suicidal ideation among undergraduates in Nigeria. This study would add to existing literature on depression and suicidal ideation among undergraduates in Nigeria which will assist in creating awareness and act as a reference document for researchers, health planners, policy makers and individuals who are concerned with the burden of depression and suicide on public health.

## Materials and methods

### Study area

Lagos State is located in the Southwestern region of Nigeria, on the Bight of Benin [28]. It lies approximately on longitude 20 42’E and 32 2’E respectively, and between latitude 60 22’N and 60 2’N. Its territorial extent and political jurisdiction encompass the city of Lagos and the four administrative divisions of Ikeja, Ikorodu, Epe and Badagry collectively referred to as IBILE, covering an area of 3,577 sq. km. which represents 0.4% of Nigeria’s territorial land mass of 923,773 sq. km [28]. Although Lagos State is the smallest state in Nigeria, it has the highest population in Nigeria [28]. According to the National Census conducted in 2006, Lagos State was reported to have a population of 9,013,534 out of the total country’s population of 140,003,542 [28]. However, according to the United Nations-Habitat and international development estimates, Lagos State was said to have about 24.6 million inhabitants in 2015 [28]. Tertiary institutions in Lagos state include; Lagos State University, University of Lagos, National Open University, Yaba College of Technology, Lagos State Polytechnic, Federal College of Education, Akoka amongst others. The study was conducted in Lagos State University and Lagos State Polytechnic.

Lagos State University is a multi-campus University having three campuses [29]. The main campus is located at Ojo, and the others at Epe (where the faculty of Engineering is located) and Ikeja (where the college of medicine is located) [29]. The University caters to about 18,000 students and offers courses for diploma, undergraduate and postgraduate levels [29]. The university has seven faculties which are the Faculty of Arts, Faculty of Education, Faculty of Engineering, Faculty of Law, Faculty of Management Sciences, Faculty of Science and Faculty of Social Sciences [29]. The university also has a College of Medicine, School of Communication, School of Transport and Postgraduate School [29].

Lagos State Polytechnic currently has three campuses located in Isolo, Surulere and Ikorodu with the Ikorodu campus being its permanent site [30]. Lagos state polytechnic has six schools namely school of management and business study, school of agriculture, school of technology, school of engineering, school of environmental studies, school of applied sciences and school of liberal and communication studies. Each school has its component department.

### Study design

The study design was a descriptive cross-sectional design to assess depression and suicidal ideation among undergraduates in the state tertiary institutions in Lagos, Nigeria.

### Eligibility criteria

#### Inclusion criteria

Only full-time students were invited to participate in the study.

#### Exclusion criteria

Students who were ill and were not present in class as at the time of the study.

### Sample size determination

The minimum sample size was determined using the Cochran formula: n = Z^2^pq/d^2^. Where Z = standard normal deviate (1.96), p = proportion of success or prevalence, q = proportion of failure = (1-p), d = tolerable margin of error (0.05) and n = minimum required sample size in population > 10,000. Using a prevalence of depression of 58.2% from a similar study in the northern part of Nigeria [31]. An estimated sample size of 373 was gotten and this value was doubled to 747 because the study was conducted among students in two tertiary institutions in Lagos. To compensate for cases of improperly completed and filled questionnaires or withdrawal from the study by any of the selected respondents, the sample size was rounded up to 780.

### Sampling technique and data collection tools

A multistage sampling technique was used to select respondents for this study. A pre-tested self-administered questionnaire was used to collect data from the respondents. The questionnaire consisted of four (A-D) sections.

SECTION A: This section contained questions that assessed sociodemographic parameters of the respondents.

SECTION B: This section was used to assess the ability of the respondents to correctly identify depression. A case vignette highlighting features of depression according to the DSM-V criteria was used for this study. Case vignettes used in studies to assess depression literacy among adolescents in Nigeria [27], as well as undergraduates [9], served as templates for developing the case vignette for this study. The vignette was about a university student named Bola who had features suggestive of depressive disorder. It was then followed by multiple choice question to assess what they believe is wrong with Bola in the case vignette. The options included ‘I don’t know’, ‘nothing is wrong with her’, spiritual problem, depression, stress, nervous breakdown, anxiety, emotional problem, substance abuse and bipolar disorder.

To remove bias in the selection of options, the project topic on the questionnaire was renamed to ‘Mental health among undergraduates in Lagos State Tertiary Institutions’. This allowed the respondents to be objective in their selection of their answer for this section.

SECTION C: This section of the questionnaire assessed the presence of depression and suicidal ideation among the respondents. The Patient-Health Questionniare-9(PHQ-9) was used to assess depressive symptoms. It is a 9-question, multi-choice scale used to assess the presence and severity of depression based on the DSM-V criteria. The questionnaire has been used in similar studies [14, 19] and has been validated among university students in Nigeria [14]. Item 9 of the scale assesses suicidal ideation by asking if the respondent thinks he would be better off dead or hurting himself.

SECTION D: This section assessed possible risk factors for depression. Factors such as low self-esteem, traumatic life events, perceived academic performance, academic satisfaction, troubles with romantic life, alcohol and substance abuse were assessed in the questionnaire.

The questions were administered in the English Language.

### Data analysis

Data was checked to ensure completeness and data from completed questionnaires were entered and analysed using SPSS version 27. Frequencies were calculated and analysed data was presented in frequency tables and figures. Association between variables was done using chi-square and level of statistical significance was set at two-tailed probability less than 5% (0.05).

### Scoring system

**Section C** uses the PHQ-9 to assess presence of depression and suicidal ideation. 9 questions were asked and 0 mark was awarded for the option ‘not at all’, 1 mark for ‘several days’, 2 marks for ‘more than half the days’ and 3 marks for ‘nearly every day’. Marks were added and total score of 1–4 indicates minimal depression, 5–9 indicates mild depression, 10–14 for moderate depression, 15–19 for moderately severe and 20–27 for severe depression.

Respondents with a score of 10 and above (PHQ≥ 10) were classified as depressed as done in other similar studies [10, 19, 32]. This is because respondents with scores of 1–9 do not have symptoms severe enough to be labelled as depressed. This would prevent misclassification of respondents with minimal and mild symptoms as depressed.

Suicidal ideation was assessed using the last question in the PHQ-9 scale.

**Section D**: Self-esteem was assessed using the Rosenberg Self-Esteem Scale. Respondents that picked the options “Strongly Disagree” were awarded 0 point, “Disagree” 1 point, “Agree” 2 points, and “Strongly Agree” 3 points. Items 10, 13, 14, 16, 17 were reversed scored. Total score ranges from 0–30. Using the mean of 15, scores below 15 indicate low self-esteem.

Alcohol disorder was assessed using the Alcohol Use Disorder Identification Test (AUDIT) scale [33]. It is a 10-question scale that was developed by the WHO to screen for alcohol disorder. Total score ranges from 0–40. A score of 20 and above is indicative of alcohol dependence.

### Ethical consideration

Ethical approval for this study was obtained from the Health Research and Ethics Committee of the Lagos University Teaching Hospital. Respondents were informed of the purpose of the study and written informed consent was obtained from the respondents prior to the administration of the questionnaire. Respondents were informed of their rights to withdraw at any time from the study and were not coerced into participation. Privacy of the respondents was protected as they were not required to write their names or addresses on the questionnaire and results gotten from the questionnaires were strictly handled with confidentiality.

## Results

A total of 780 questionnaires were administered, 750 were adequately completed and analysed making a response rate of 96.2%. Eighteen of the questionnaires were not completely filled and 12 questionnaires were not returned, thereby excluding them from further analysis.

Table 1 shows the Socio-demographic characteristics of the respondents. About 48.3% of the respondents were undergraduate from Lagos State University while the rest were from the polytechnic. The age range of 20–24 has the highest (65.5%) frequency and the mean age of the respondents was 21.5 ± 2.7 years. More than half of the respondents (54%) were females. More of the respondents, 70.3% were Christians and almost all the respondents were single (98.1%). The source of income for majority of the respondents was parents (72.8%).

**Table 1:** Socio-demographic characteristics of the respondents (n = 750).

Variables	Frequency (n)	Percentage (%)
**Institutions**		
Lagos State University	388	48.3
Lagos State Polytechnic	362	51.7
**Age group in years**		
15–19	181	24.1
20–24	491	65.5
25–29	73	9.7
30–34	5	0.6
Mean (S.D)	21.5±2.7	
**Sex**		
Female	405	54.0
Male	345	46.0
**Marital status**		
Married	14	1.9
single	736	98.1
**Religion**		
Christianity	527	70.3
Islam	223	29.7
Source of income/allowance*		
Parents	546	72.8
Self-employed	155	20.6
Employed	30	4.0
Partner	19	2.5

Based on the respondents answer to the vignette, Table 2 reveals that less than half (47.6%) of the respondents were able to correctly label Bola’s character as depressed. The options chose by the remaining 52.4% of the respondents were I don’t know (8.9%), stress (10.5%), emotional problems (14.1%), nervous breakdown (5.9%), nothing was wrong with her (1.6%) and anxiety (2.9%). Table 3 shows that respondents with PHQ≥10, that is from moderate to severe depressive symptoms were considered as depressed. The prevalence of depression in this study was 22.5% and the prevalence of suicidal ideation among the respondents was found to be 21.6%.

**Table 2:** Respondents ability to identify depression in the case vignette (n = 750).

Vignette label	Frequency (n)	Percentage (%)
Anxiety	22	2.9
Bipolar disorder	20	2.7
Depression	357	47.6
Emotional problem	106	14.1
Nervous breakdown	44	5.9
Stress	79	10.5
Substance abuse	16	2.1
Spiritual problem	17	2.3
There is nothing wrong with her	12	1.6
I don’t know	67	8.9

**Table 3:** Knowledge and presence of depression and suicidal ideation among the respondents (n = 750).

Variables	Frequency (n)	Percentage (%)
Knowledge of depression		
Good	357	47.6
Poor	393	52.4
Presence of depression		
No (PHQ < 10)	581	77.5
Yes (PHQ ≥10)	169	22.5
Presence of suicidal ideation		
No	588	78.4
Yes	162	21.6

**Fig 1:**
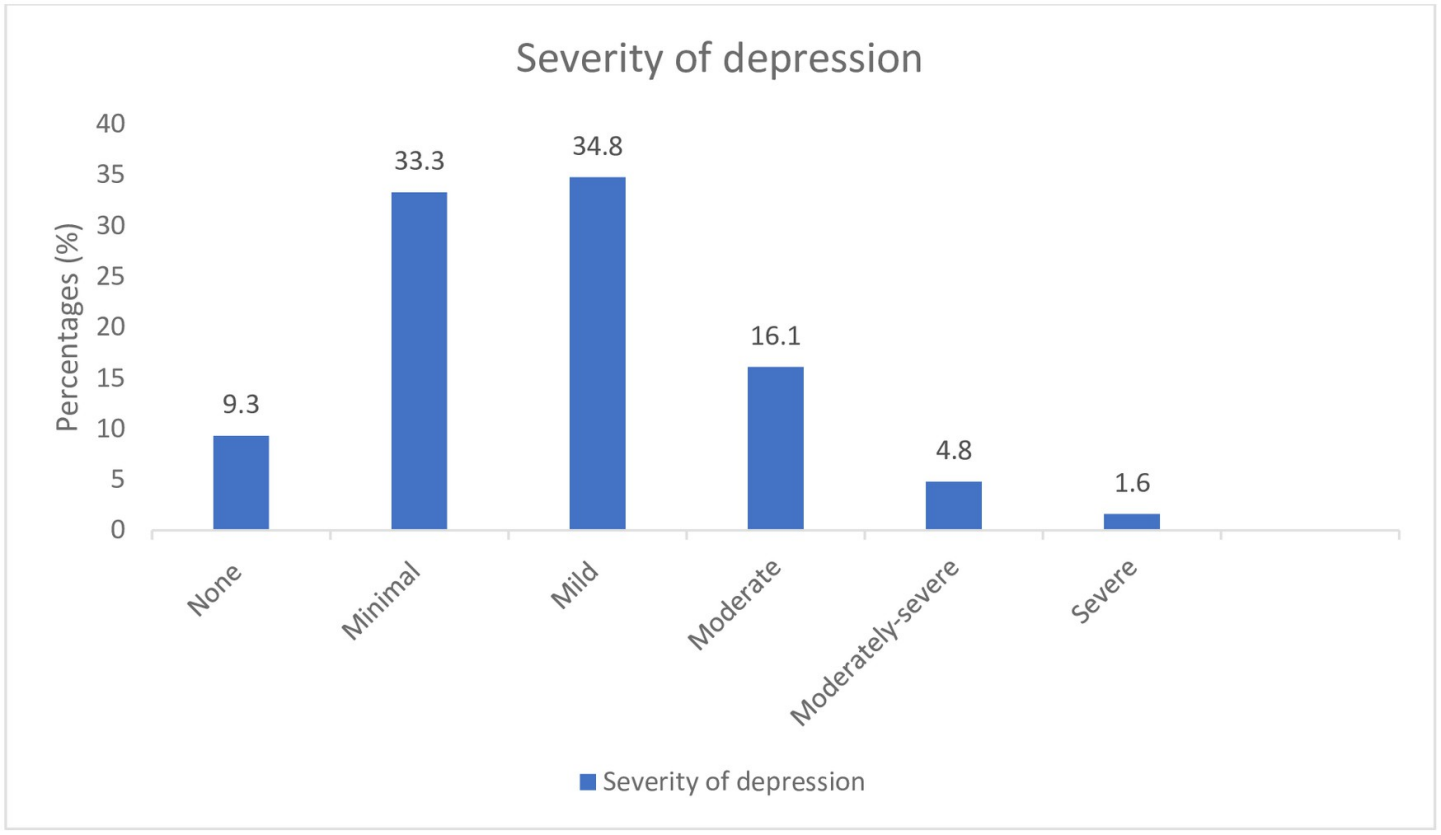
Severity of depression among the respondents.

Fig 1 reveals the distribution of the depressive symptoms among the respondents. These include; no depression (9.3%), minimal depression (33.3%), mild depression (34.8%), moderate depression (16.1%), moderately severe depression (4.8%) and severe depression (1.6%).

Table 4 describes the association between the socio-demographic characteristics and knowledge of depression among the respondents. Source of income of the respondents was the only characteristics that was statistically significant with the knowledge of depression among the respondents.

**Table 4:** Association between sociodemographic characteristics and knowledge of depression.

Variables	knowledge of depression		X^2^	P-value
	Good 357 (47.6%)	Poor 393 (52.4%)		
**Age group in years**				
15–19	87 (48.1)	94 (51.9)	5.266	0.662
20–24	236 (48.1)	255 (51.9)		
25–29	31 (42.5)	42 (57.5)		
30–34	3 (60.0)	2 (40.0)		
**Sex**				
Female	197 (48.6)	208 (51.4)	0.383	0.536
Male	160 (46.4)	185 (53.6)		
**Marital status**				
Married	6 (42.9)	8 (57.1)		
single	351 (47.7)	385 (52.3)	0.129	0.720
**Religion**				
Christianity	259 (49.1)	268 (50.9)		
Islam	98 (43.9)	125 (56.1)	1.699	0.192
Source of income/allowance*				
Parents	276 (50.5)	270 (49.5)		
Self-employed	63 (40.6)	92 (59.4)		
Employed	13 (43.3)	17 (56.6)		
Partner	4 (21.1)	15 (78.9)	21.298	*0*.*018*

**Fig 2:**
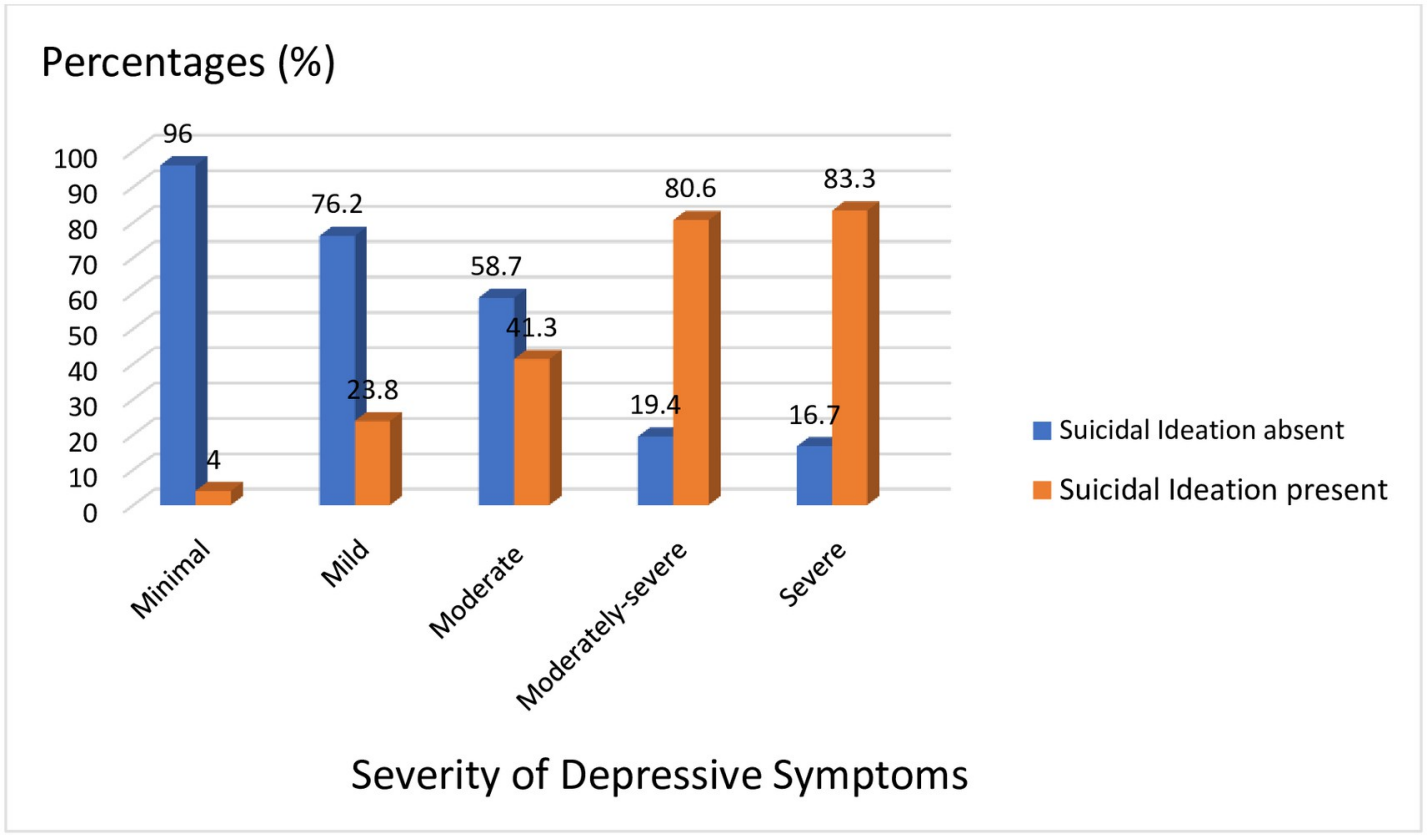
Severity of depressive symptoms and suicidal ideation.

Fig 2 shows an association between severity of depressive symptoms and prevalence of suicidal ideation. Among the respondents with minimal depressive symptoms, prevalence of suicidal ideation was among only 4% of them. But among the respondents with moderate depressive symptoms, suicidal ideation was present among about 41.3% and prevalence of suicidal ideation was 83.3% among respondents with severe depressive symptoms.

Table 5 shows the association between the risk factors and presence of depression among the respondents. Positive history of sexual assault, bullying and being hit by a partner were statistically significantly associated with depression. Other risk factors that were significantly associated with depression among the respondents were low self-esteem, poor academic performance, alcohol dependence.

**Table 5:** Association between the risk factors and depression among respondents.

Variables	Presence of depression		X^2^	P-value
	No 581 (77.5%)	Yes 169 (22.5%)		
**History of traumatic life events**				
**History of sexual assault**				
No	511 (78.7)	138 (21.3)		
Yes	70 (69.3)	31 (30.7)	4.452	*0*.*035*
**History of bullying**				
No	368 (84.6)	67 (15.4)		
Yes	213 (67.6)	102 (32.4)	30.172	*<0*.*001*
**History of being hit by partner**				
No	418 (81.2)	97 (18.8)		
Yes	163 (69.4)	72 (30.6)	12.879	*<0*.*001*
**Recent loss of a loved one**				
No	398 (78.8)	107 (21.2)		
Yes	183 (74.7)	62 (25.3)	1.603	0.206
**Relationship status**				
**Currently in a romantic relationship**				
No	273 (79.4)	71 (20.6)		
Yes	308 (75.9)	98 (24.1)	1.306	0.253
Recently break-up from a romantic relationship				
No	421 (79.4)	109 (20.6)		
Yes	160 (72.7)	60 (27.3)	4.006	0.045
Satisfied with your current relationship status				
No	164 (75.9)	52 (24.1)		
Yes	417 (78.1)	117 (29.9)	0.413	0.512
**Respondents self-esteem**				
Low (score <15)	35 (43.8)	45 (56.2)		
High (score ≥15)	546 (81.5)	124 (18.5)	58.321	*<0*.*001*
**Respondents academic performance**				
Perceived academic performance				
Poor	156 (69.6)	68 (30.4)		
Good	425 (80.8)	101 (19.2)	14.185	*0*.*003*
Satisfaction with academic performance				
Satisfied	265 (81.8)	59 (18.2)		
Unsatisfied	316 (74.2)	110 (25.8)	6.108	*0*.*013*
**Respondents intake of recreational drugs**				
Intake of recreational drugs				
No	541 (78.7)	146 (21.3)		
Yes	40 (63.5)	23 (36.5)	7.695	*0*.*013*
Respondents alcohol dependence				
Dependent (score ≥ 20)	5 (38.5)	8 (61.5)		
Non-dependent (score <20)	576 (78.2)	161 (21.8)	9.274	*0*.*001*

Table 6 shows the association between the risk factors and suicidal ideation among the respondents. Risk factors that were statistically significantly associated with suicidal ideation were Positive history of sexual assault, bullying and being hit by a partner, break-up from a romantic relationship, low self-esteem, poor academic performance, alcohol dependence and presence of depression.

**Table 6:** Association between the risk factors and suicidal ideation among respondents.

Variables	Suicidal ideation		X^2^	P-value
	No 588 (78.4%)	Yes 162 (21.6%)		
**History of traumatic life events**				
**History of sexual assault**				
No	517 (79.7)	132 (20.3)		
Yes	71 (70.3)	30 (29.7)	4.525	*0*.*033*
**History of bullying**				
No	361 (83.0)	74 (17.0)		
Yes	227 (72.1)	88 (27.9)	12.877	*< 0*.*001*
**History of being hit by partner**				
No	416 (80.8)	99 (19.2)		
Yes	172 (73.2)	63 (26.8)	5.483	*0*.*019*
**Recent loss of a loved one**				
No	403 (79.8)	102 (20.2)		
Yes	185 (75.5)	60 (24.5)	1.794	0.180
**Relationship status**				
**Currently in a romantic relationship**				
No	274 (79.7)	70 (20.3)		
Yes	314 (77.3)	92 (22.7)	0.587	0.443
Recently break-up from a romantic relationship				
No	429 (80.9)	101 (19.1)		
Yes	159 (72.3)	61 (27.7)	6.902	*0*.*009*
Satisfied with your current relationship status				
No	161 (74.5)	55 (25.5)		
Yes	427 (80.0)	107 (20.0)	2.673	0.102
**Respondents self-esteem**				
Low (score <15)	32 (40.0)	48 (60.0)		
High (score ≥15)	556 (83.0)	114 (17.0)	77.977	*<0*.*001*
**Respondents academic performance**				
Perceived academic performance				
Poor	156 (72.4)	68 (27.6)		
Good	432 (83.8)	94 (16.2)	17.728	*0*.*001*
Satisfaction with academic performance				
Satisfied	271 (83.6)	53 (16.4)		
Unsatisfied	317 (74.4)	109 (25.6)	9.256	*0*.*002*
**Respondents intake of recreational drugs**				
Intake of recreational drugs				
No	552 (80.3)	135 (19.7)		
Yes	36 (57.1)	37 (42.9)	18.352	*< 0*.*001*
Respondents alcohol dependence				
Dependent (score ≥ 20)	5 (38.5)	8 (61.5)		
Non-dependent (score <20)	583 (79.1)	154 (20.9)	18.352	*<0*.*001*
**Presence of depression**				
No (PHQ < 10)	510 (87.8)	71 (12.2)		
Yes (PHQ ≥10)	78 (46.2)	91 (53.8)	133.955	*<0*.*001*

## Discussion

The proportion of the undergraduate in this study with good knowledge of depression was 47.6%. The prevalence of depression and suicidal ideation among the respondents was 22.5% and 21.6% respectively. Findings from this study also revealed the risk factors associated with depression and suicidal ideation, these risk factors are; sexual assault, bullying, being hit by a partner, self-esteem, academic performance and alcohol dependence. Presence of depression was significantly associated with suicidal ideation.

Less than half (47.6%) of the respondents in this study were able to correctly identify depression. Similar studies with poor knowledge of depression were conducted in Sri-Lanka (17.4%) [9] and South-eastern and western part of Nigeria with 4.8% and 10.4% respectively [27, 34]. However, in a similar study conducted among undergraduates in the United States, 63% of the respondents were able to correctly identify depression [35]. Similarities were also seen in a study done in Portugal, where 61% of the respondents were able to correctly identify depression [36]. The reason for the disparity in the results could be as a result of the use of close-ended questions in this study, whereas other studies with lower proportions of respondents with good knowledge used open-ended questions which left no room for guessing.

Furthermore, the reason for the less than satisfactory ability of the respondents to identify depression among the respondents (Table 2) can be examined by looking at the vignette label used by the respondents to identify the character in the case vignette. The three most common mislabels for depression from this study were emotional problem (14.1%), stress (10.5%) and nervous breakdown (5.9%). About 9% of the respondents selected ‘I don’t know’. This is similar to a study done in Portugal where the three most common mislabels for depression from the vignette were stress, emotional problem and nervous breakdown [36]. A similar study done in Sri-Lanka, also revealed stress as the most common mislabel of depression [9]. Another study done in Vietnam revealed that stress was the most selected option, even more than depression [37]. The respondents in this current study, were not able to clearly differentiate symptoms of stress, nervous breakdown and emotional problem (and the other selected options in the vignette), from the symptoms of depression. Hence, the reasons for these mislabels could be attributed to not only the poor knowledge of the symptoms of depression, but also poor knowledge of the manifestations of depressive symptoms.

The prevalence of depression among the respondents was 22.5%. This is slightly lower than the prevalence of a similar study done among undergraduates in Ethiopia where the prevalence was 26.8% [38]. Other similar studies with similar results were done in Sri-Lanka and the United States where the prevalence was 22.3% and 29.3% respectively [9, 11]. A study done in Nigeria in 2018 among undergraduates revealed the prevalence of depression to be 58.2% [31]. The reason for this high figure can be attributed to the fact that the study used the PHQ-9, just like this current study, however, respondents who had minimal- mild depressive symptoms were labelled depressed. If the respondents with minimal-mild depressive symptoms were excluded, then the prevalence of depression in that study was 21.2%, which is similar to this current study. It is important to note that the prevalence in this current study is however much higher than another study done in Nigeria where the prevalence of depression was 8.3% [14]. This finding suggests that there has been an increase in the prevalence of depression among undergraduates in Nigeria.

The prevalence of suicidal ideation in this study was 21.6%. This was similar to the prevalence of suicidal ideation among students in Botswana [39]. In contrast, the prevalence was slightly higher than that found in a similar study conducted in Ethiopia where the prevalence was 19.9% [40]. Also, lower rates of 6.6%-11% were recorded in similar studies conducted in the United States [41, 42]. The finding is also much higher than the prevalence of suicidal ideation of the general population as evidenced by the mental health survey carried out in Lagos state in which the prevalence was 7.28% [43]. Therefore, more attention needs to be made towards undergraduates, as this study and other similar studies have also suggested that they are at a higher risk of having suicidal ideation compared to the rest of the population.

Depression was found to be strongly associated with suicidal ideation in this study as shown in Table 6. More than half of the respondents with depression in this study (53.8%) reported having suicidal thoughts. The strong relationship between depression and suicidal ideation has been validated in various studies [44–46]. Prevalence of suicidal ideation also increases with severity of depressive symptoms (Fig 2). These positive linear relationship between depression severity and suicidal ideation has also been found in other similar studies worldwide [44, 46–48]. The implication of this is that the more the severity of the symptoms of depression in an individual, the more likely the individual is to have suicidal thoughts.

Some risk factors were statistically significantly associated with either depression or suicidal ideation. However, some risk factors were statistically significantly associated with both depression and suicidal ideation. Risk factors that were statistically associated with depression and suicidal ideation in this study were bullying, low self-esteem, alcohol dependence, poor academic performance, dissatisfaction with academic performance, hit by partner and sexual assault.

Association between bullying and depression and suicidal ideation has been validated in previous studies [49, 50]. In one of these studies, the proportion of youths with depression increased with the frequency of bullying, with 14.8% of those who were frequently bullied coming down with depression, compared with 5.5% of those not bullied who had depression [50]. The deleterious effects of being bullied continues long after the incident itself, and makes the individual vulnerable to mental health problems. The implication of this is that the trauma of being bullied (even as a child), is a major risk factor for the development of depression in adulthood.

Low self-esteem was found to be strongly associated with not only depression, but also suicide in this study (Tables 5 and 6). This relationship has been validated in also similar studies [51, 52]. This significant relationship between low self-esteem and depression and suicidal ideation, can be as a result of the strong negative feelings about one’s self or feeling like a failure, expressed by individuals with low self-esteem. Such strong feelings can have negative effects on mental health, and as evidenced in this study, and play a major contribution to development of depression and suicidal ideation.

Alcohol dependence was also found to be associated with both depression and suicidal ideation in this study. Similarly, hazardous alcohol use has also been found to be related to both depression [53–55], and suicide [18], in multiple studies. Also, frequent use of recreational drugs was found to be both related to depression and suicide. This relationship has also been validated in multiple similar studies on depression and suicide [38].

Poor academic performance was statistically significantly associated with suicidal ideation, unlike other studies [6, 12], there was also a significant association between it and depression. Non-satisfaction with romantic status and romantic break-up was found to be associated with suicidal ideation but not depression in this study. However, a similar study revealed a relationship between break-up and depression [9]. Nevertheless, the feeling of abandonment or loneliness felt after a break-up can explain why it is a risk factor for developing suicidal thoughts.

Unlike other studies in which the female gender was associated with depression [14, 56], findings in this study did not reveal any relationship between the female gender and depression. This is in line with similar studies that suggest that there is an equal risk of having depression in both genders [53]. Like other studies [57, 58], sexual assault and being hit by a partner were significantly associated with depression and suicidal ideation (Tables 5 & 6).

## Conclusion and recommendations

A strong relationship was found between depression and suicidal ideation indicating that people with depression are at a high risk of having suicidal ideation. Risk factors that were associated with depression and suicidal ideation were bullying, low self-esteem, intake of recreational drugs, alcohol dependence, poor academic performance, sexual assault and being hit by a partner.

The following recommendations were made based on the findings from this study. There should be increase awareness of the causes and manifestations of depression among undergraduates and the general public by the Federal Ministry of Health in partnership with the Non-Governmental Organisations and school administrations. Periodical screening for depression and suicidal ideation should be done in universities to enable early detection of these problems and also early intervention strategies to solve it. There should be provision of effective and user-friendly counselling services in higher institutions for students by the school administration and awareness should be made to the public on suicide hotlines by the Federal Ministry of Health.
